# Supplementary material for: Manipulation of Subcortical and Deep Cortical Activity in the Primate Brain Using Transcranial Focused Ultrasound Stimulation
Source: Neuron. 2019 Mar 20;101(6):1109–1116.e5. doi: 10.1016/j.neuron.2019.01.019 (PMC6520498; doi:10.1016/j.neuron.2019.01.019)
Supplement: Document S1. Figures S1–S4 [file mmc1.pdf]

Neuron, Volume *101*

Supplemental Information

Manipulation of Subcortical and Deep Cortical Activity in the Primate Brain Using Transcranial Focused Ultrasound Stimulation

Davide Folloni, Lennart Verhagen, Rogier B. Mars, Elsa Fouragnan, Charlotte Constans, Jean-François Aubry, Matthew F.S. Rushworth, and Jérôme Sallet

SUPPLEMENTAL INFORMATION

Manipulation of subcortical and deep cortical activity in the primate brain using transcranial focused ultrasound stimulation

Davide Folloni, Lennart Verhagen, Rogier B. Mars, Elsa Fouragnan, Charlotte Constans, Jean-François Aubry, Matthew F.S. Rushworth, Jérôme Sallet

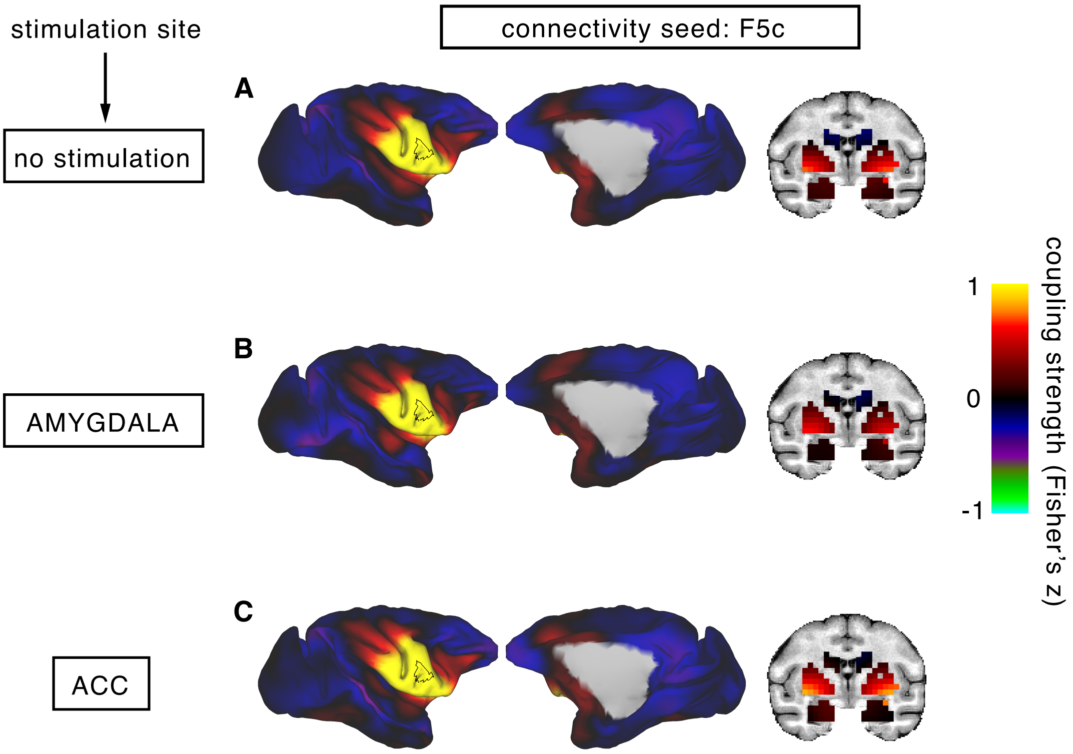

Figure S1. Whole-brain functional connectivity between stimulated and not stimulated areas with the rest of the brain, related to Figure 2

Panels A, B, and C show activity coupling between a control area, the caudal ventral premotor area F5c (outlined in black), and the rest of the brain in no stimulation/control condition (**A**), after amygdala TUS (**B**), and after ACC TUS (**C**). Hot colors indicate positive coupling (Fisher's z). Compared to a no stimulation condition (A), neither amygdala TUS nor ACC TUS (B, C) affected the whole-brain coupling activity of F5c which has weak anatomical connections with ACC and amygdala.

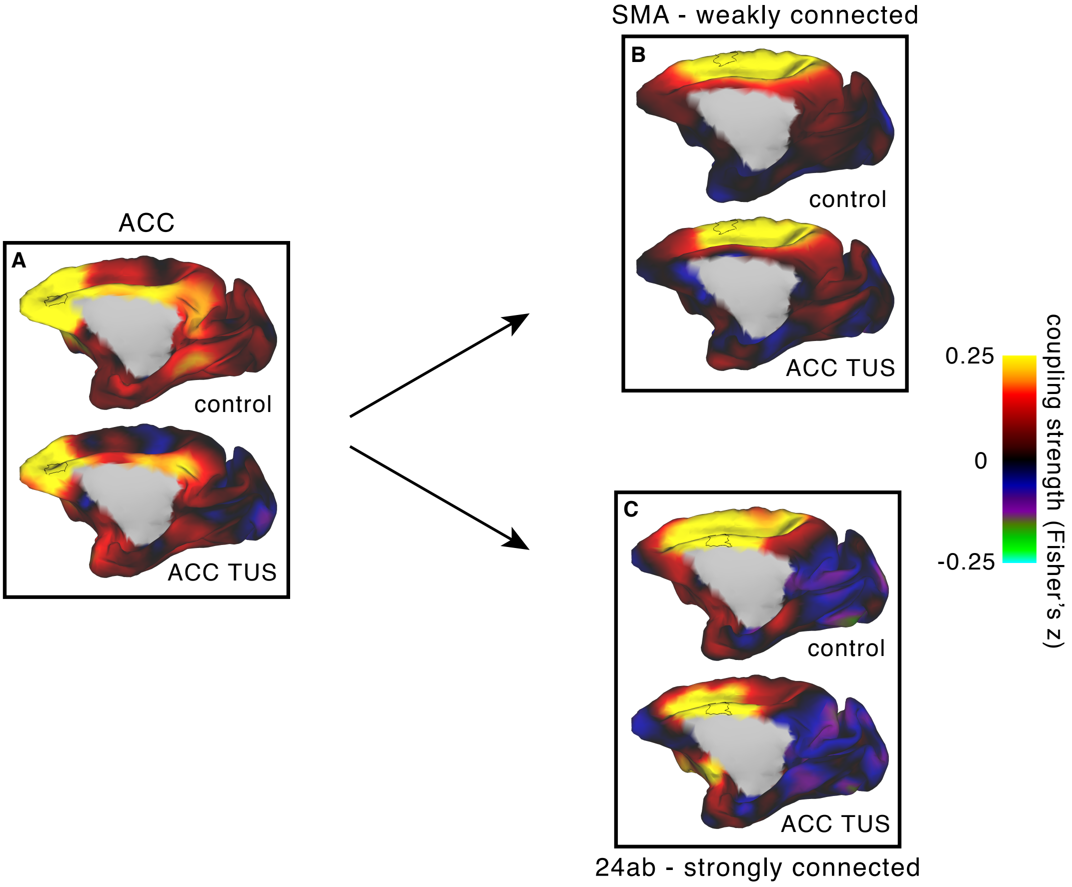

Figure S2. Effects of TUS on regions outside the target area are mediated by the strength of anatomical connectivity rather than a result of spatial proximity, related to Figure 3

Whole-brain activity coupling in control and ACC TUS conditions for ACC (**A**) and two areas at an equal Euclidian distance from the ACC target region but differing in their anatomical connectivity strength with the stimulated area: SMA (**B**) and area 24ab (**C**). Hot colors indicate positive coupling (Fisher's z). In SMA, which is less strongly connected to ACC, there were no changes (B) in the way in which its activity was coupled with that in other brain areas. This was in contrast with changes in activity coupling of area 24ab (C), which is more strongly connected to the ACC target compared to SMA (B).

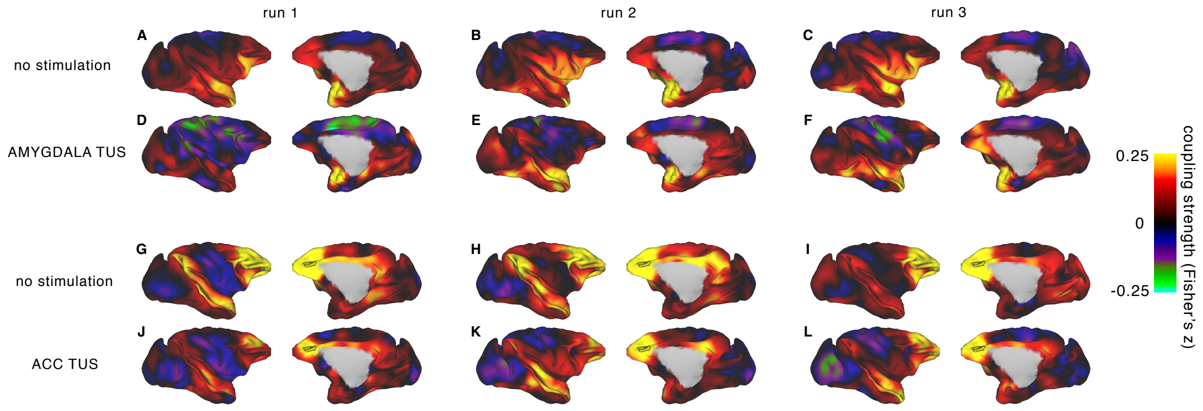

run 1
run 2
run 3
A
B
C
no stimulation
D
E
F
AMYGDALA TUS
G
H
I
no stimulation
J
K
L
ACC TUS
0.25
0
-0.25
coupling strength (Fisher's z)

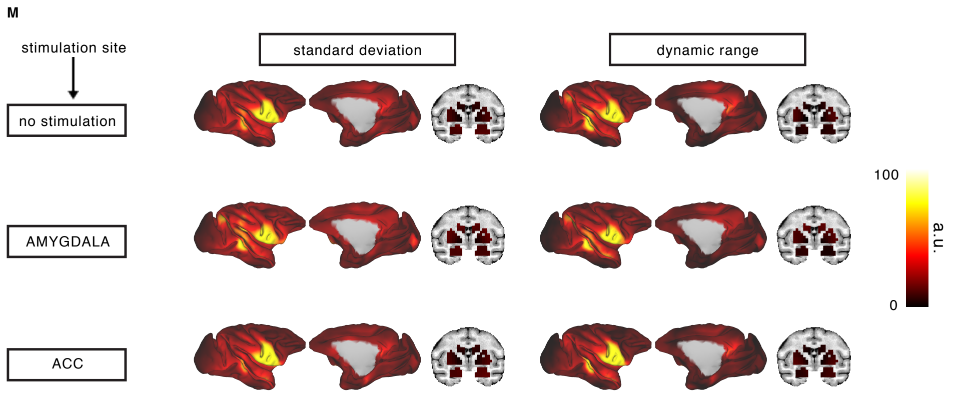

M
stimulation site
standard deviation
dynamic range
no stimulation
AMYGDALA
ACC
100
0
a.u.

Figure S3. Temporal changes of TUS effects on amygdala and ACC functional coupling, related to Figures 2-3

Amygdala and ACC functional coupling across 3 consecutive runs is displayed after no stimulation (amygdala: **A**,**B**,**C**; ACC: **G**,**H**,**I**) and TUS (amygdala: **D**,**E**,**F**; ACC: **J**,**K**,**L**). TUS effects on the whole-brain coupling of each stimulated region persisted throughout the full length of scanning. Interestingly, TUS effects seem to show slightly decrease over the three runs with amygdala and ACC functional coupling resembling more their correspondent coupling in the no stimulation condition. (**M**) Gross indices of signal variability, time after induction, and physiological state related to anesthesia levels revealed no major effects of TUS condition. Temporal variability of the BOLD signal - a proxy for both the signal amplitude and the noise level - were similar across the control state and after either amygdala or ACC TUS. The anesthesia levels as indexed by expired isoflurane concentrations were well matched between conditions (see STAR Methods), the delay between sedation and data collection was on average comparable between amygdala TUS (2.00h) and the control (2.38h) sessions (amygdala TUS versus control: $F(1,34)=2.7654$, $p=0.10552$) but slightly shorter in the ACC TUS (1.44h; versus control: $F(1,31)=9.5537$, $p=0.0041946$). However, despite differences in the effects of ACC and amygdala TUS on functional connectivity, amygdala and ACC TUS sessions were very similar in duration ($F(1,19)=2.6863$, $p=0.11767$). Furthermore, there were no differences in any measurements of physiological parameters indexing the depth of anesthesia including expired isoflurane ($F(1.41)=0.37451$, $p=0.68995$), heart rate ($F(1,41)=1.8382$, $p=0.17198$), and respiration rate ($F(1,41)=0.032232$, $p=0.96831$) in control, amygdala, and ACC sessions.

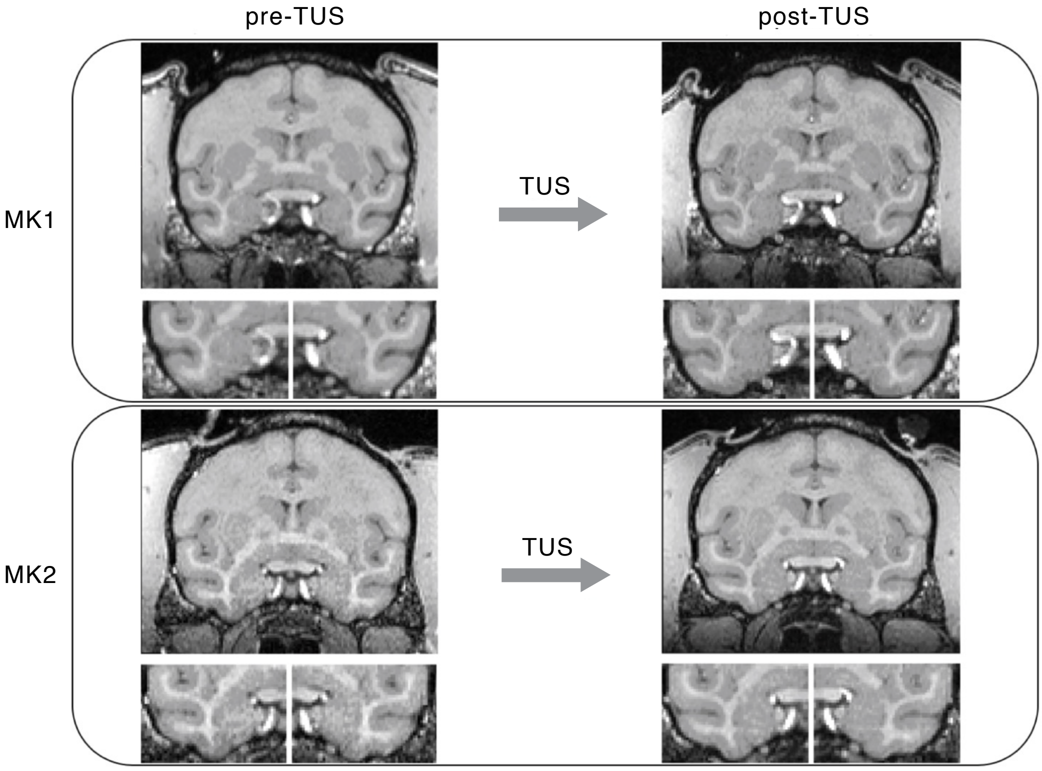

Figure S4. Lack of effects of TUS on brain tissue, related to STAR Methods

Coronal T1-weighted images collected before and after TUS (in this example targeted bilaterally to the amygdala) representing the stimulated brain area. Neither structural changes nor evidence of transient edema were found following TUS targeted to the amygdala bilaterally in two exemplar animals.
