# Supplementary material for: Manipulation of Subcortical and Deep Cortical Activity in the Primate Brain Using Transcranial Focused Ultrasound Stimulation
Source: Neuron. 2019 Mar 20;101(6):1109–1116.e5. doi: 10.1016/j.neuron.2019.01.019 (PMC6520498; doi:10.1016/j.neuron.2019.01.019)
Supplement: Document S2. Article plus Supplemental Information [file mmc2.pdf]

Report

Neuron

Manipulation of Subcortical and Deep Cortical Activity in the Primate Brain Using Transcranial Focused Ultrasound Stimulation

Highlights

- Ultrasound stimulation exerts regionally specific neural effects in primates
- It can be used to alter activity even in subcortical and deep cortical areas
- After stimulation, activity in a brain area is less related to that of its network
- The effect lasted for more than 1 h and was not mediated by auditory confounds

Authors

Davide Folloni, Lennart Verhagen, Rogier B. Mars, ..., Jean-François Aubry, Matthew F.S. Rushworth, Jérôme Sallet

Correspondence

davide.folloni@psy.ox.ac.uk (D.F.), lennart.verhagen@psy.ox.ac.uk (L.V.), jerome.sallet@psy.ox.ac.uk (J.S.)

In Brief

Ultrasound can be used to modulate activity in deep brain areas. After stimulation, activity in the targeted brain area becomes less coupled to its network. Effects are specific to the stimulation site, long-lasting, and not due to auditory confounds.

Folloni et al., 2019, Neuron *101*, 1109–1116
March 20, 2019 © 2019 Elsevier Inc.
https://doi.org/10.1016/j.neuron.2019.01.019

CellPress

CellPress

Manipulation of Subcortical and Deep Cortical Activity in the Primate Brain Using Transcranial Focused Ultrasound Stimulation

Davide Folloni,[1,2,7,8,*] Lennart Verhagen,[1,2,7,*] Rogier B. Mars,[2,3] Elsa Fouragnan,[1,4] Charlotte Constans,[5] Jean-François Aubry,[6] Matthew F.S. Rushworth,[1,2,7] and Jérôme Sallet[1,2,7,*]
[1]Wellcome Centre for Integrative Neuroimaging (WIN), Department of Experimental Psychology, University of Oxford, Oxford OX1 3SR, UK
[2]Wellcome Centre for Integrative Neuroimaging (WIN), Centre for Functional MRI of the Brain (FMRIB), Nuffield Department of Clinical Neurosciences, John Radcliffe Hospital, University of Oxford, Oxford OX3 9DU, UK
[3]Donders Institute for Brain, Cognition and Behaviour, Radboud University Nijmegen, 6525 HR Nijmegen, the Netherlands
[4]School of Psychology, University of Plymouth, Plymouth PL4 8AA, UK
[5]Physics for Medicine Paris, Inserm, ESPCI Paris, CNRS, PSL Research University, Univ Paris Diderot, Sorbonne Paris Cité, Paris 75012, France
[6]Physics for Medicine Paris, Inserm, ESPCI Paris, CNRS, PSL Research University, Paris 75012, France
[7]These authors contributed equally
[8]Lead Contact
*Correspondence: davide.folloni@psy.ox.ac.uk (D.F.), lennart.verhagen@psy.ox.ac.uk (L.V.), jerome.sallet@psy.ox.ac.uk (J.S.)
https://doi.org/10.1016/j.neuron.2019.01.019

SUMMARY

The causal role of an area within a neural network can be determined by interfering with its activity and measuring the impact. Many current reversible manipulation techniques have limitations preventing their application, particularly in deep areas of the primate brain. Here, we demonstrate that a focused transcranial ultrasound stimulation (TUS) protocol impacts activity even in deep brain areas: a subcortical brain structure, the amygdala (experiment 1), and a deep cortical region, the anterior cingulate cortex (ACC, experiment 2), in macaques. TUS neuromodulatory effects were measured by examining relationships between activity in each area and the rest of the brain using functional magnetic resonance imaging (fMRI). In control conditions without sonication, activity in a given area is related to activity in interconnected regions, but such relationships are reduced after sonication, specifically for the targeted areas. Dissociable and focal effects on neural activity could not be explained by auditory confounds.

INTRODUCTION

To establish the functional role of a brain area, it is necessary to examine the impact of disrupting or altering its activity. It has recently been proposed that this might be accomplished with low-intensity focused transcranial ultrasound stimulation (TUS) (Tufail et al., 2011; Yoo et al., 2011). While a TUS impact on behavior has been described (Deffieux et al., 2013), little is known about its impact on neural activity and if its effects persist after stimulation is terminated. We show here that in the macaque, TUS modulates neural activity and does so even in subcortical nuclei such as the amygdala and deep cortical regions such as anterior cingulate cortex (ACC). Moreover, we demonstrate a protocol that exerts an "offline" effect that lasts for an extended period of tens of minutes after an initial stimulation period of 40 s. This extended period of action is important, because it means that its neural effect substantially outlasts any potential direct acoustic or somatosensory effects that might occur during the stimulation period itself (Guo et al., 2018; Sato et al., 2018). We also confirm this by showing that the stimulation protocol was not associated with any similarly sustained impact on the activity of the auditory system.

Finally, we demonstrate that a considerable degree of focality is possible with TUS. The peak and extent of the TUS neuromodulatory effect closely matched those of the ultrasonic intensity as estimated by simulations of the acoustic wave propagation. When TUS is applied to amygdala, its impact is most apparent in amygdala rather than in more distal regions or those between the stimulation cone and the target area. The same is true of ACC TUS; its impact is most apparent in ACC, where the acoustic intensity is highest. The focal impact of offline TUS in deep brain structures may underlie the specific patterns of behavioral impairment recently reported when the same protocol was used in awake behaving animals (Fouragnan et al., 2018).

RESULTS

Stimulation of Deep Brain Structure and Resting-State fMRI Recording

On each session of TUS application, a 40-s train of pulsed ultrasound (250 kHz) comprising 30-ms bursts of ultrasound every 100 ms was directed to the target brain region using a single-element transducer in conjunction with a region-specific coupling cone filled with degassed water. To control for any confounds resulting from concomitant ultrasound stimulation and neural signal recording (Guo et al., 2018; Sato et al., 2018), recordings of neural activity only begun approximately 30 min after

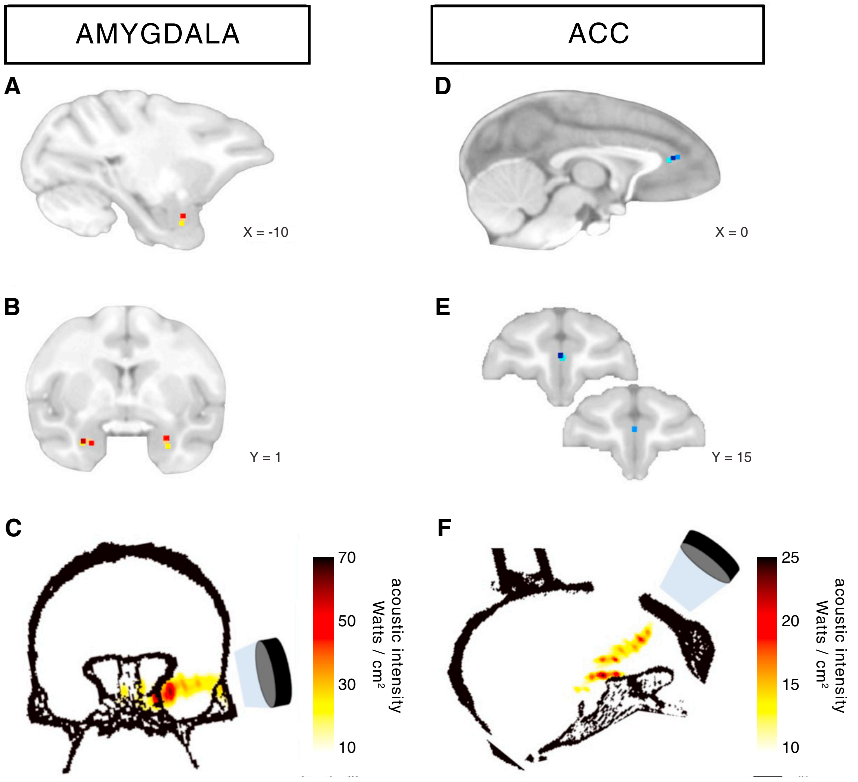

Figure 1. Stimulation Targets

(A–F) Stimulation target position is shown for each individual animal (colored dots) on sagittal and coronal views for TUS targeted at amygdala (A and B) and ACC (D and E). Acoustic intensity field (W/cm^2) generated by the ultrasound beam in the brain is shown for one example animal per TUS target, amygdala (C) and ACC (F). The target position can be delineated with accuracy in all animals in (A), (B), (D), and (E) by using each individual's own MRI scan. As a result, the activity and functional connectivity of the target areas can be examined accurately in each animal. However, some slight imprecision in the estimation in the acoustic intensity maps in (C) and (F) may occur; this is because group average targets are used in conjunction with the computed tomography X-ray scan of a single individual during the modeling.

the end of TUS application when any potential auditory or somatosensory effects of stimulation were dissipated. We therefore refer to this stimulation protocol as an "offline" protocol.

Frameless stereotaxic neuronavigation was used to position the transducer over the target brain area, taking into consideration the focal depth of the sonication (Figure 1; experiment 1: amygdala, n = 4; experiment 2: ACC, n = 3; relatively deep brain regions known to be interconnected and co-active during similar cognitive processes such as social cognition) (Munuera et al., 2018; Noonan et al., 2014). A single train was applied sequentially to each amygdala in experiment 1 and to the midline structure, ACC, in experiment 2.

The impact of TUS was determined by examining brain activity over an 80-min period starting ~30 min after the 40-s stimulation train began (STAR Methods). Activity was recorded not just from the stimulated site but from across the entire brain using functional magnetic resonance imaging MRI (fMRI). fMRI data from the stimulated animals were compared with data from an additional group of control individuals (n = 9) that had received no TUS. Note that depth of anesthesia and the delay between sedation induction and data acquisition were similar between the TUS and control groups (0.7%–0.8% and 0.7%–1% range of expired isoflurane concentration, 1.53 and 2.38 h, respectively; STAR Methods; Figure S3M). fMRI data were acquired at 3 T under isoflurane anesthesia and processed using established tools and protocols (Verhagen et al., 2019; STAR Methods). The anesthesia protocol has previously been shown to preserve regional functional connectivity measurable with fMRI (Sallet et al., 2013; Neubert et al., 2015).

Effects of TUS on Subcortical Neural Activity in the Amygdala

To examine the spatial specificity of TUS effects and to investigate the capacity of TUS to stimulate subcortical structures we investigated its effects on the coupling of amygdala activity with activity in other brain areas. Even at rest in the control state, blood-oxygen-level-dependent (BOLD) activity in one area is correlated with BOLD activity in other areas, and such relationships are most prominent when the areas are monosynaptically connected, although some residual connectivity is mediated by indirect connections (O'Reilly et al., 2013). The pattern of activity coupling for any given area reflects its unique constellation of projections and interactions, sometimes called its "connectivity fingerprint" (Passingham et al., 2002).

In the control state, amygdala activity was coupled with activity in cingulate, ventral prefrontal, and orbitofrontal cortex, striatum, and the anterior temporal lobe (Figures 2A and 2G). To limit the risk of false positive and negative results, we focused our analysis on a limited set of brain regions known to be interconnected with the amygdala based on previous studies (Neubert et al., 2014, 2015; Sallet et al., 2013) and compared the overall patterns or "fingerprint" of coupling (amygdala TUS versus control) using cosine similarity metrics in a nonparametric statistical framework (see STAR Methods for details).

The amygdala's activity coupling was significantly changed after amygdala TUS (nonparametric permutation test, p = 0.0020; Figures 2B and 2G). A whole-brain quantitative analysis revealed that this effect of amygdala TUS was most apparent in the amygdala and not anywhere else in the brain (see Focality of TUS Effect; Figure 3A).

A second way to establish the specificity of TUS effects within the network is to examine whether the amygdala connectivity effects seen after amygdala TUS are found after ACC TUS. This was not the case; ACC TUS left most of amygdala's coupling pattern unaffected (nonparametric permutation test,

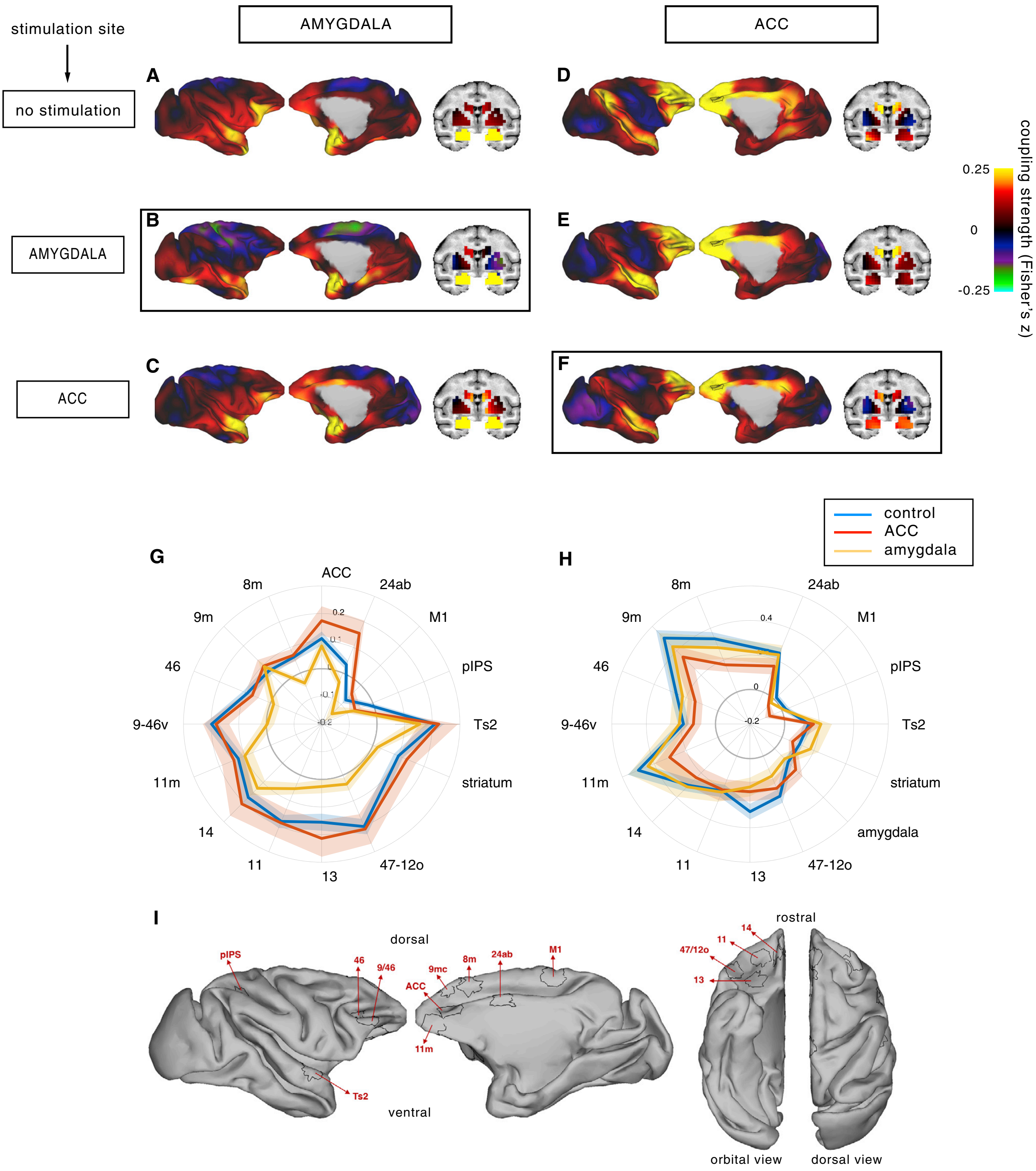

Figure 2. Amygdala and ACC Functional Coupling Changes after Stimulation

(A–C) Activity coupling between amygdala (in yellow on the coronal view) and the rest of the brain in the no stimulation (control) condition (A), after amygdala TUS (B), and after ACC TUS (C).

(D–F) Activity coupling between ACC (outlined in black) and the rest of the brain in the no stimulation (control) condition (D), after amygdala TUS (E), and after ACC TUS (F). Hot colors indicate positive coupling (Fisher's z). Functional connectivity from TUS-targeted regions is highlighted by black boxes. Each type of TUS had a selective effect on the stimulated area; amygdala coupling was strongly changed by amygdala TUS only (B), and ACC coupling was strongly changed by ACC TUS only (F).

(legend continued on next page)

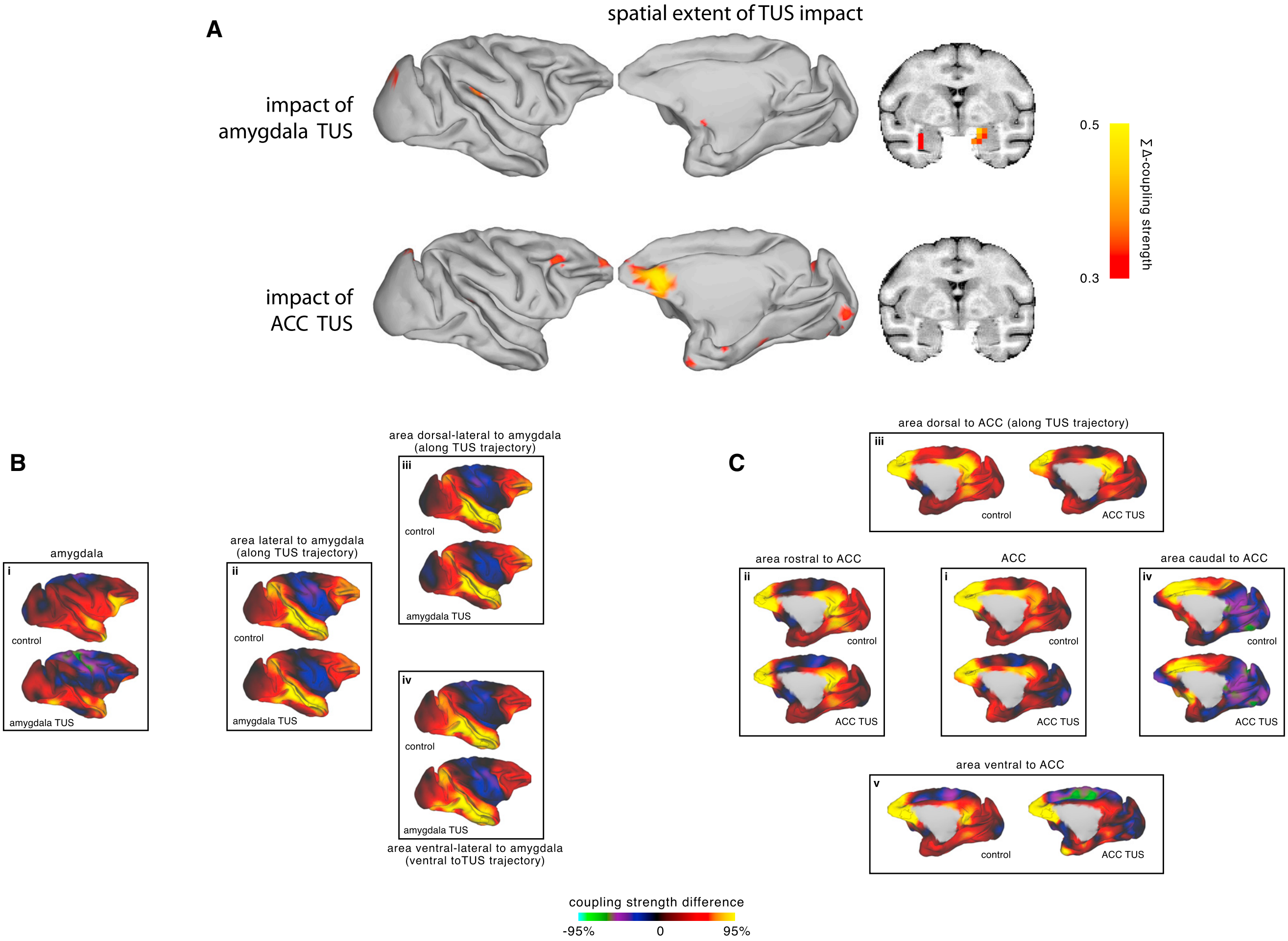

Figure 3. Spatial Extent of the TUS Neuromodulatory Effect and Its Impact on Areas Neighboring the Stimulated Region

(A) Amplitude and spatial extent of the impact of amygdala TUS (top row) and ACC TUS (bottom row) on the coupling of each point in the brain with the same set of *a-priori*-defined areas used in Figures 2G and 2H. Hot colors indicate a strong decrement in coupling after TUS compared to the control state (summed delta Fisher's z). The effect of TUS on activity coupling was restricted to the amygdala after amygdala TUS (top row) and to the ACC and regions immediately ventral along the ultrasound trajectory following ACC TUS (bottom row).

(B) The whole-brain coupling of the amygdala target region (i, also shown in Figure 2) and regions along (ii and iii) or immediately surrounding the trajectory of the ultrasound stimulation beam (iv) in the control condition and after amygdala TUS. There were no major changes in the coupling of these off-target regions and the rest of the brain.

(C) The whole-brain coupling of the ACC target region (i, also shown in Figure 2) and surrounding regions near the ACC target (ii–v) in both the ACC TUS and control conditions. Some changes in coupling can be seen along the stimulation trajectory in the area just ventral to the target (v) and also in an area that is unlikely to have been hit directly by the ultrasound beam (iv). These areas are strongly anatomically connected with the targeted area.

Seed regions in (B) and (C) are indicated with black outlines.

p = 0.1346; Figures 2C and 2G), although not surprisingly, ACC TUS led to alteration in amygdala's coupling with ACC.

Finally, to further establish the nature of amygdala TUS effects within the network, we investigated the activity coupling patterns of five further control areas. We investigated three regions adjacent to the amygdala and found their functional connectivity was unaltered (Figure 3B). We also examined an area with a very distinct constellation of projections (ventral premotor area F5c) and again found no change (Figure S1). Similarly, analyses of the temporal variability of the BOLD signal (Figure S3M) did not

(G) Connectivity fingerprint representation of the strength of activity coupling between amygdala and other brain areas in control animals (blue), after amygdala TUS (yellow), and after ACC TUS (red).

(H) Activity coupling between ACC and the rest of the brain in control animals (blue), after ACC TUS (red), and after amygdala TUS (yellow). Each type of TUS had a selective effect on the stimulated area; amygdala coupling was strongly affected by amygdala TUS (the yellow line is closer to the center of the panel than the blue line), and ACC coupling was strongly disrupted by ACC TUS (the red line is closer to the center of the panel than the blue line). SEM is indicated by shading around each line.

(I) The regions of interest constituting the fingerprints depicted on lateral, medial, orbital, and dorsal views.

reveal TUS-induced changes in signal amplitude or noise level, suggesting that TUS effects are specific to changes in signal coupling of the stimulated region.

Below, we explain additional control analyses that confirmed that the TUS effect could not have been mediated via auditory cortex.

Effects of TUS on Deep Cortical Neural Activity in ACC

To examine the specificity of TUS effects further and investigate the capacity of TUS to stimulate deep cortical structures, we investigated the effects of ACC TUS on ACC activity. In control animals, ACC activity at rest was coupled with activity in strongly connected areas: dorsal, lateral, and orbital prefrontal cortex (PFC), frontal pole, and mid and posterior cingulate (Figures 2D and 2H). After ACC TUS, the ACC coupling pattern was altered (nonparametric permutation test, $p = 0.0210$; Figures 2F and 2H). A parsimonious interpretation is that normally, the activity that arises in ACC is a function of the activity in the areas that project to it, but this is no longer the case when ACC's activity is artificially driven or diminished by TUS. Because these interactions with other areas determine the information ACC receives from elsewhere in the brain and the influence it exerts over other areas, ACC TUS should alter ACC's computation and induce specific changes in behavior (Fouragnan et al., 2018).

Similar to the analyses of spatial extent of amygdala TUS effects, we quantified the change in coupling induced by ACC TUS not only in ACC itself but also for every point in the brain. This analysis revealed that ACC TUS affected primarily the ACC (see Focality of TUS Effect; Figure 3A).

The specificity and selectivity of the effects are further underscored by the results observed when mapping the coupling pattern of areas interconnected with the stimulated ACC region. First, we examined the activity coupling pattern of the amygdala, an area with which ACC is monosynaptically interconnected (Amaral and Price, 1984; Van Hoesen et al., 1993) and functionally coupled (Neubert et al., 2015). Not surprisingly, there was some evidence that amygdala-ACC coupling had changed as a function of ACC TUS, as had coupling with a third area, caudal orbitofrontal cortex, with which both ACC and amygdala are strongly interconnected. However, other aspects of the amygdala's coupling pattern were relatively unaltered by ACC TUS; although there was a trend in the nonparametric permutation test for amygdala connectivity to change after ACC TUS ($p = 0.0744$; Figures 2C and 2H), it was clear that there was a significant difference between ACC and amygdala TUS effects (nonparametric permutation test, $p = 0.0428$).

Just as amygdala TUS did not affect the connectional profile of F5c, a region outside the interconnected network of the stimulated areas, ACC TUS also did not affect F5c's coupling (Figure S1). Again, below, we explain additional control analyses that confirmed that the TUS effect could not have been mediated via the auditory cortex.

The spatial and connectional specificity of the observed effects make it unlikely that the TUS induced modulations were mediated by general physiological effects, such as those related to anesthesia level and duration (see STAR Methods and Figure S3M for details).

Focality of TUS Effect

To examine the focality of the TUS effect on activity coupling, two additional sets of analyses were conducted. The first set of analyses assessed the impact of TUS on the connectivity fingerprint not only for the target areas but also for every point in the brain resulting in "heatmaps" of TUS impact, while the second set focused in detail on the areas surrounding the target areas or located between the stimulation cone and the target area (Figures 3 and S2).

In the first set of analyses, following amygdala TUS, the strongest neuromodulatory effects were observed in the amygdala itself, and only in the amygdala (Figure 3A, top row). Following ACC TUS, the extent of the neuromodulation was limited to the ACC and regions immediately ventral to it along the ultrasound beam (Figure 3A, bottom row). In fact, the spatial maps of TUS impact on activity coupling are strikingly in correspondence with the spatial maps of estimated sonication intensity (Figures 1C and 1F). This correspondence is specific and sensitive; it includes particulars of the wave propagations, such as how the ultrasound wave targeted at amygdala reflects on the basal bone, while in the ACC TUS condition, considerable acoustic energy is also deposited immediately ventral to the target along the trajectory, partly due to sound waves reflecting on the orbital bone.

To further qualify and examine the extent of the ultrasonic intervention, in the second set of analyses, we assessed the impact of TUS on whole-brain activity coupling of control areas surrounding the sonication target or along the trajectory of the ultrasound beam (Figures 3B and 3C). Confirming the spatial maps of TUS impact (Figure 3A), and matching the estimated contours of the acoustic intensity (Figure 1F), there were no major changes in the activity coupling of areas situated between the target and the transducer (Figure 3B, ii and iii; and Figure 3C, iii), while as estimated following ACC TUS, there were some changes in the connectional profile of a region along the stimulation trajectory just ventral to the ACC target (Figure 3C, v).

Finally, we examined whether areas outside the directly sonicated region but strongly connected to it might exhibit a network-derived effect of TUS. Changes to the connectional profile of an area just posterior (Figure 3C, iv) to the target region could be suggestive of such a network effect. Indeed, additional analyses of areas sharing the same spatial proximity but lacking the same anatomical connectedness confirmed these changes were likely due to the anatomical connections this region shared with the target area (Figure S2).

Putative Auditory Effects of Offline TUS

It has recently been suggested that the impact of TUS on neural activity is mediated by its auditory impact (Guo et al., 2018; Sato et al., 2018). Several considerations suggest that it might not be possible to explain away the current findings as the result of an auditory artifact. First, the auditory impact of TUS is likely a function of specific features of its frequency and pulse type, especially of the frequency used to modulate the ultrasonic carrier wave. Second, the auditory stimulation associated with the TUS application ceased after the sonication, but the neural activity measurements were initiated tens of minutes later. Third, TUS of each area, ACC and amygdala, had specific effects that were distinct from one another.

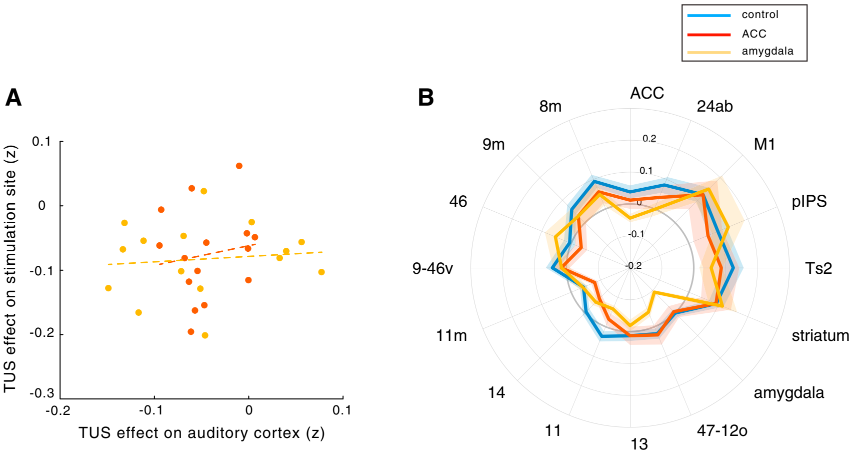

Figure 4. Effect of Amygdala and ACC TUS on the Functional Coupling of Primary Auditory Cortex

(A) ACC TUS (red line) had no effect on the functional coupling of A1. Amygdala TUS (yellow line) affected the relationship between A1's activity and activity in several areas that are linked to the A1 via the amygdala, including the amygdala itself, lateral orbitofrontal cortex area 47/12o, and ACC.

(B) Mediation via the auditory cortex cannot explain the effects seen after either amygdala (yellow) or ACC (red) TUS.

Nevertheless, we also carried out a fourth line of inquiry and examined whether it is plausible that an auditory effect could have mediated the effects of TUS on amygdala and ACC. To quantify this probability, we correlated any TUS effects on primary auditory cortex (A1) connectivity with TUS effects on the targeted regions (Figure 4A). TUS effects on the auditory cortex after both amygdala ($r = 0.1084$, $p = 0.7007$) and ACC ($r = 0.1474$, $p = 0.6000$) sonication are unrelated to the TUS effects at each target site and are therefore unlikely to have mediated effects seen at the stimulation sites. However, it is possible that TUS over amygdala or ACC had an impact on A1 connectivity that was separate from its impact on the stimulated sites themselves (Figure 4B). While A1 connectivity is not impacted by ACC TUS (Figure 4B; nonparametric permutation test, $p = 0.6871$), amygdala TUS did have a significant impact on A1 connectivity (Figure 4B; nonparametric permutation test, $p = 0.0002$). Closer inspection revealed that this was due to a diminution solely in A1's interactions with the amygdala itself and two areas with which the amygdala is itself strongly connected with: ACC and orbitofrontal cortex. Differential effects of ACC and amygdala TUS on A1 connectivity might be driven by some direct, albeit weak, connections of amygdala with A1 (Yukie, 2002). Similarly, given amygdala's strong connections to ACC and orbitofrontal cortex, it is perhaps not surprising that amygdala sonication might affect A1's interactions with them. Importantly, these circumscribed effects on A1 connectivity are not predictive of the effects elsewhere. In summary, the alteration seen in the A1 fingerprint is a poor match to the alteration seen in the amygdala fingerprint after amygdala TUS or in the ACC fingerprint after ACC TUS.

DISCUSSION

In these investigations, we combined TUS with resting-state fMRI to examine the impact of modulating activity in subcortical and deep cortical areas of the primate brain. Experiments 1 and 2 revealed dissociable effects of amygdala and ACC TUS. The dissociable nature of the effects and the fact that they were observed more than 1 h after the 40-s stimulation period suggests they are not mediated by the stimulation's auditory impact (Guo et al., 2018; Sato et al., 2018). In each case, the effects were apparent as reductions in activity coupling between the stimulated area and other regions with which it is normally interconnected; after TUS, a brain area's activity appears to be driven less by activity in the areas with which it is connected and more by the artificial modulation induced by TUS.

Any impact that TUS exerts on the auditory system is likely to depend on the precise details of the sonication frequency, pulse and modulation frequency, and pulse shape and might be specific to other features of the preparation, such as anesthesia level (Airan and Butts Pauly, 2018). Here, we employed an ultrasound frequency of 250 kHz that we pulse modulated at 10 Hz; as such, we ensured that both the ultrasound carrier wave and the wave envelope frequency were well outside of the macaque hearing range. This can be contrasted against more conventional protocols where the ultrasound is pulse modulated at ~1 kHz, within the audible range of both rodents and primates. Moreover, the offline stimulation protocol we employed also made it less likely that the auditory system was stimulated at the time that neural activity was recorded; neural activity was only measured many minutes after the cessation of a 40-s period of TUS.

Our aim in the current study was to examine whether TUS can modulate neural activity. The results obtained demonstrate that TUS can exert a relatively focal and circumscribed impact on neural activity. However, as a consequence of using a recording technique that is sensitive to a number of neurophysiological processes, it was not possible to establish the precise nature of the neurophysiological process that mediated the fMRI signal effects that we observed. It is possible that TUS may act not simply by immediately inducing or reducing activity in neurons but by modulating their responsiveness to other neural inputs; thus, its effect may have been more easily detected by an analysis strategy such as the current one that focused on measuring the relationship between activity in the stimulated area and elsewhere. As with other repetitive neurostimulation protocols, it is also possible that TUS's offline effects are partly driven by the induction of plastic changes, with long-term-potentiation or depression-like characteristics, and again, this might have implications for how its effects are best detected.

Several molecular mechanisms describing how low-intensity ultrasound stimulation modulates neuronal activity have been suggested. However, recent investigations on the interactions between sound pressure waves and brain tissue suggest that

ultrasound primarily exerts its modulatory effects through a mechanical action on cell membranes, notably affecting ion channel gating (Kubanek et al., 2016; Tyler et al., 2008). While the precise mechanisms are being determined (Fomenko et al., 2018; Tyler et al., 2018), the current results suggest that TUS may be suitable as a tool for focal manipulation of activity in many brain areas in primates. Specifically, they show that TUS may even be used to manipulate activity in subcortical structures in monkeys.

TUS's capacity to stimulate subcortical and deep cortical areas in primates therefore opens the prospect of advanced noninvasive causal brain mapping. To date, noninvasive manipulation of brain activity in humans can be done reversibly only by using neuromodulation methods such as transcranial magnetic stimulation and transcranial current stimulation. However, the spatial resolution of some of these techniques is limited (Polanía et al., 2018). Even more critically, application of these techniques is constrained to the surface of the brain, as their efficacy falls off rapidly with depth.

Before it becomes possible to use repetitive TUS to study the human brain in a routine manner, a number of considerations must be borne in mind. It will be important to establish the safety of the technique. In another recent study, we have shown that TUS of the type used here causes no permanent damage to tissue on histological analysis (Verhagen et al., 2019). Structural MRI scans collected shortly after TUS in the present study showed no evidence of transient edema (Figure S4). While such results are encouraging, further studies may be needed to establish if this remains true even after a greater number of TUS sessions, after TUS sessions of longer duration, or after TUS at a greater intensity. Care may need to be taken with the assessment of each new protocol that is devised. It should also be noted that its neural effects may be sustained over a period of time that is substantially longer than in many laboratory experiments (Verhagen et al., 2019); care will therefore need to be taken in deciding when a human participant might leave the laboratory and travel home. In addition, under some circumstances, sonication appears to impact the meninges (Verhagen et al., 2019), and the full nature of this impact may need to be established. This not only has safety implications but also suggests that the impact of TUS on a brain area is best assessed by comparison to the impact of TUS on an appropriate control site.

In summary, based on the results reported here, TUS can be used to transiently and reversibly alter neural activity in subcortical and deep cortical areas with high spatial specificity. To date, it is the most promising neuromodulatory technique to reach areas deep below the dorsolateral surface of the brain in a minimally invasive and focal manner, thereby providing it with the potential for causally mapping brain functions within and across species. While it may currently lack the capacity to target specific neurons, as do some optogenetic and chemogenetic techniques (Yizhar et al., 2011), it may provide a method for investigating brain areas that may make it suitable for use with primate species, which are rarely investigated with such techniques, even though many brain areas are particularly well developed or only present in primates (Passingham and Wise, 2012). With care, it may even be possible to employ offline TUS protocols in investigations of human brain function.

STAR★METHODS

Detailed methods are provided in the online version of this paper and include the following:

- KEY RESOURCES TABLE
- CONTACT FOR REAGENT AND RESOURCE SHARING
- EXPERIMENTAL MODEL AND SUBJECT DETAILS
- METHOD DETAILS
  - Ultrasound stimulation
  - Macaque MRI acquisition
  - Macaque MRI preprocessing
- QUANTIFICATION AND STATISTICAL ANALYSIS
  - Macaque rs-fMRI connectivity analysis
  - Macaque rs-fMRI statistical inference
- DATA AND SOFTWARE AVAILABILITY

SUPPLEMENTAL INFORMATION

Supplemental Information includes four figures and can be found with this article online at https://doi.org/10.1016/j.neuron.2019.01.019.

ACKNOWLEDGMENTS

Funding for this study was provided by a Wellcome Senior Investigator Award (WT100973AIA), the MRC (program grants MR/P024955/1 and G0902373), a Wellcome Trust Henry Dale Fellowship (105651/Z/14/Z), the Wellcome Trust (UK Grant 105238/Z/14/Z), The Wellcome Centre for Integrative Neuroimaging (203139/Z/16/Z), the BBRSC (grant BB/N019814/1), the NWO (grant 452-13-015), the Bettencourt Schueller Foundation, and the Agence Nationale de la Recherche under the program ‘‘Future Investments’’ (ANR-10-EQPX-15).

AUTHOR CONTRIBUTIONS

D.F. and J.S. designed the experiment; D.F. and J.S. collected the data; D.F., L.V., M.F.S.R., and J.S. analyzed the data; L.V. and R.B.M. contributed analysis tools; C.C. and J.-F.A. contributed to the ultrasound modeling; and D.F., L.V., E.F., M.F.S.R., J.-F.A., and J.S. wrote the manuscript.

DECLARATION OF INTERESTS

The authors declare no competing interests.

Received: July 18, 2018
Revised: November 29, 2018
Accepted: January 14, 2019
Published: February 11, 2019

REFERENCES

Airan, R.D., and Butts Pauly, K. (2018). Hearing out ultrasound neuromodulation. Neuron *98*, 875–877.

Amaral, D.G., and Price, J.L. (1984). Amygdalo-cortical projections in the monkey (Macaca fascicularis). J. Comp. Neurol. *230*, 465–496.

Constans, C., Deffieux, T., Pouget, P., Tanter, M., and Aubry, J.-F. (2017). A 200-1380-kHz quadrifrequency focused ultrasound transducer for neurostimulation in rodents and primates: transcranial in vitro calibration and numerical study of the influence of skull cavity. IEEE Trans. Ultrason. Ferroelectr. Freq. Control *64*, 717–724.

Dallapiazza, R.F., Timbie, K.F., Holmberg, S., Gatesman, J., Lopes, M.B., Price, R.J., Miller, G.W., and Elias, W.J. (2018). Noninvasive neuromodulation and thalamic mapping with low-intensity focused ultrasound. J. Neurosurg. *128*, 875–884.

Deffieux, T., Younan, Y., Wattiez, N., Tanter, M., Pouget, P., and Aubry, J.-F. (2013). Low-intensity focused ultrasound modulates monkey visuomotor behavior. Curr. Biol. *23*, 2430–2433.

Fomenko, A., Neudorfer, C., Dallapiazza, R.F., Kalia, S.K., and Lozano, A.M. (2018). Low-intensity ultrasound neuromodulation: an overview of mechanisms and emerging human applications. Brain Stimul. *11*, 1209–1217.

Fouragnan, E.F., Chau, B.K., Folloni, D., Kolling, N., Verhagen, L., Klein-Flugge, M., Tankelevitch, L., Papageorgiou, G.K., Aubry, J.-F., Sallet, J., and Rushworth, M.F. (2018). The macaque anterior cingulate cortex translates counterfactual choice value into actual behavioral change. bioRxiv. https://doi.org/10.1101/336917.

Glasser, M.F., Sotiropoulos, S.N., Wilson, J.A., Coalson, T.S., Fischl, B., Andersson, J.L., Xu, J., Jbabdi, S., Webster, M., Polimeni, J.R., et al.; WU-Minn HCP Consortium (2013). The minimal preprocessing pipelines for the Human Connectome Project. Neuroimage *80*, 105–124.

Guo, H., Hamilton, M., 2nd, Offutt, S.J., Gloeckner, C.D., Li, T., Kim, Y., Legon, W., Alford, J.K., and Lim, H.H. (2018). Ultrasound produces extensive brain activation via a cochlear pathway. Neuron *98*, 1020–1030.e4.

Jenkinson, M., Beckmann, C.F., Behrens, T.E.J., Woolrich, M.W., and Smith, S.M. (2012). FSL. Neuroimage *62*, 782–790.

Kubanek, J., Shi, J., Marsh, J., Chen, D., Deng, C., and Cui, J. (2016). Ultrasound modulates ion channel currents. Sci. Rep. *6*, 24170.

Margulies, D.S., Vincent, J.L., Kelly, C., Lohmann, G., Uddin, L.Q., Biswal, B.B., Villringer, A., Castellanos, F.X., Milham, M.P., and Petrides, M. (2009). Precuneus shares intrinsic functional architecture in humans and monkeys. Proc. Natl. Acad. Sci. USA *106*, 20069–20074.

Mars, R.B., Verhagen, L., Gladwin, T.E., Neubert, F.-X., Sallet, J., and Rushworth, M.F.S. (2016). Comparing brains by matching connectivity profiles. Neurosci. Biobehav. Rev. *60*, 90–97.

Munuera, J., Rigotti, M., and Salzman, C.D. (2018). Shared neural coding for social hierarchy and reward value in primate amygdala. Nat. Neurosci. *21*, 415–423.

Neubert, F.-X., Mars, R.B., Thomas, A.G., Sallet, J., and Rushworth, M.F.S. (2014). Comparison of human ventral frontal cortex areas for cognitive control and language with areas in monkey frontal cortex. Neuron *81*, 700–713.

Neubert, F.-X., Mars, R.B., Sallet, J., and Rushworth, M.F.S. (2015). Connectivity reveals relationship of brain areas for reward-guided learning and decision making in human and monkey frontal cortex. Proc. Natl. Acad. Sci. USA *112*, E2695–E2704.

Noonan, M.P., Sallet, J., Mars, R.B., Neubert, F.X., O'Reilly, J.X., Andersson, J.L., Mitchell, A.S., Bell, A.H., Miller, K.L., and Rushworth, M.F.S. (2014). A neural circuit covarying with social hierarchy in macaques. PLoS Biol. *12*, e1001940.

O'Reilly, J.X., Croxson, P.L., Jbabdi, S., Sallet, J., Noonan, M.P., Mars, R.B., Browning, P.G.F., Wilson, C.R.E., Mitchell, A.S., Miller, K.L., et al. (2013). Causal effect of disconnection lesions on interhemispheric functional connectivity in rhesus monkeys. Proc. Natl. Acad. Sci. USA *110*, 13982–13987.

Passingham, R.E., and Wise, S.P. (2012). The Neurobiology of the Prefrontal Cortex: Anatomy, Evolution, and the Origin of Insight, First Edition. Oxford Psychology Series (Oxford University Press).

Passingham, R.E., Stephan, K.E., and Kötter, R. (2002). The anatomical basis of functional localization in the cortex. Nat. Rev. Neurosci. *3*, 606–616.

Polanía, R., Nitsche, M.A., and Ruff, C.C. (2018). Studying and modifying brain function with non-invasive brain stimulation. Nat. Neurosci. *21*, 174–187.

Reveley, C., Gruslys, A., Ye, F.Q., Glen, D., Samaha, J., E Russ, B., Saad, Z., K Seth, A., Leopold, D.A., and Saleem, K.S. (2017). Three-dimensional digital template atlas of the macaque brain. Cereb. Cortex *27*, 4463–4477.

Sallet, J., Mars, R.B., Noonan, M.P., Neubert, F.-X., Jbabdi, S., O'Reilly, J.X., Filippini, N., Thomas, A.G., and Rushworth, M.F. (2013). The organization of dorsal frontal cortex in humans and macaques. J. Neurosci. *33*, 12255–12274.

Sato, T., Shapiro, M.G., and Tsao, D.Y. (2018). Ultrasonic neuromodulation causes widespread cortical activation via an indirect auditory mechanism. Neuron *98*, 1031–1041.e5.

Smith, S.M., Hyvärinen, A., Varoquaux, G., Miller, K.L., and Beckmann, C.F. (2014). Group-PCA for very large fMRI datasets. Neuroimage *101*, 738–749.

Tufail, Y., Yoshihiro, A., Pati, S., Li, M.M., and Tyler, W.J. (2011). Ultrasonic neuromodulation by brain stimulation with transcranial ultrasound. Nat. Protoc. *6*, 1453–1470.

Tyler, W.J., Tufail, Y., Finsterwald, M., Tauchmann, M.L., Olson, E.J., and Majestic, C. (2008). Remote excitation of neuronal circuits using low-intensity, low-frequency ultrasound. PLoS ONE *3*, e3511.

Tyler, W.J., Lani, S.W., and Hwang, G.M. (2018). Ultrasonic modulation of neural circuit activity. Curr. Opin. Neurobiol. *50*, 222–231.

Van Essen, D.C., and Dierker, D.L. (2007). Surface-based and probabilistic atlases of primate cerebral cortex. Neuron *56*, 209–225.

Van Hoesen, G., Morecraft, R., and Vogt, B. (1993). Connections of the monkey cingulate cortex. In Neurobiology of Cingulate Cortex and Limbic Thalamus (Birkhäuser), pp. 249–284.

Verhagen, L., Gallea, C., Folloni, D., Constans, C., Jensen, D., Ahnine, H., Roumazeilles, L., Santin, M., Ahmed, B., Lehericy, S., Klein-Flugge, M., Krug, K., Mars, R.B., Rushworth, M.F., Pouget, P., Aubry, J.-F., and Sallet, J. (2019). Offline impact of transcranial focused ultrasound on cortical activation in primates. eLife *8*, e40541.

Vincent, J.L., Patel, G.H., Fox, M.D., Snyder, A.Z., Baker, J.T., Van Essen, D.C., Zempel, J.M., Snyder, L.H., Corbetta, M., and Raichle, M.E. (2007). Intrinsic functional architecture in the anaesthetized monkey brain. Nature *447*, 83–86.

Yizhar, O., Fenno, L.E., Davidson, T.J., Mogri, M., and Deisseroth, K. (2011). Optogenetics in neural systems. Neuron *71*, 9–34.

Yoo, S.-S., Bystritsky, A., Lee, J.-H., Zhang, Y., Fischer, K., Min, B.-K., McDannold, N.J., Pascual-Leone, A., and Jolesz, F.A. (2011). Focused ultrasound modulates region-specific brain activity. Neuroimage *56*, 1267–1275.

Yukie, M. (2002). Connections between the amygdala and auditory cortical areas in the macaque monkey. Neurosci. Res. *42*, 219–229.

STAR★METHODS

KEY RESOURCES TABLE

REAGENT or RESOURCE	SOURCE	IDENTIFIER
**Chemicals, Peptides, and Recombinant Proteins**		
Isoflurane – ISOFLO 250ml	Centaur	30135687
Ketamine – Narketan 10% 10ml INJ CD(SCH4)1 1-MCD	Centaur	03120257
Midazolam – Hypnoval amps 10mg/2ml	Centaur	23191407
Atropine – Atrocare INJ 25ml	Centaur	01300236
Meloxicam – Metacam INJ 10ml 5mg/ml DOGS/CATS	Centaur	02500456
Ranitidine 50mg/2ml x5 INJ	Centaur	30294115
Saline	DPAG, University of Oxford	N/A
Formalin	DPAG, University of Oxford	N/A
SignaGel Electrode Gel	Parker Laboratories	#15-25
**Deposited Data**		
Structural and functional MRI data, anaesthesia parameters, and physiological measurements	Lennart Verhagen, Davide Folloni, Jerome Sallet	https://git.fmrib.ox.ac.uk/lverhagen/amygdala-acc-tus
**Experimental Models: Organisms/Strains**		
Macaca mulatta, 9 males, 2 females, between 4-11 years old, between 7-15 kg, socially housed	MRC, Centre for Macaques	NCBITaxon:9544
**Software and Algorithms**		
MATLAB 2017a	Mathworks	RRID: SCR_001622
FMRIB Software Library v5.0	FMRIB, WIN, Oxford, UK	RRID: SCR_002823
Connectome Workbench	The Human Connectome Project and Connectome Coordination Facility	RRID: SCR_008750
Magnetic Resonance Comparative Anatomy Toolbox	Neuroecology Lab	https://github.com/neuroecology/MrCat
**Other**		
Transducer H-115MR 250kHz SN:018	Sonic Concepts	http://sonicconcepts.com
Transducer H-115MR 250kHz SN:017	Sonic Concepts	http://sonicconcepts.com
Amplifier Model 75A250A – 75Watts – 10khz 250MHz	Amplifier Research	http://www.arworld.us
Tie Pie Handyscope HS5 SN: 32239	Tie Pie	https://www.tiepie.com/en
Brainsight frameless stereotaxic neuronavigation system	Rogue Research	RRID: SCR_009539
MRI compatible frame	Crist Instruments	http://www.cristinstrument.com/products/stereotax/stereotax-primate
four-channel phased-array coil	Windmiller Kolster Scientific	https://www.wkscientific.com/#mri-coils

CONTACT FOR REAGENT AND RESOURCE SHARING

Further information and requests for resources should be directed to and will be fulfilled by the Lead Contact, Davide Folloni (davide.folloni@psy.ox.ac.uk).

EXPERIMENTAL MODEL AND SUBJECT DETAILS

For this study, resting-state fMRI (rs-fMRI) and anatomical MRI scans were collected for 11 healthy macaques (*Macaca mulatta*, NCBITaxon:9544, nine males, two females, age: 7.3 years, weight: 10.3 kg). Resting state fMRI from four animals were acquired post amygdala TUS (experiment 1; n = 4); rs-fMRI from three animals were acquired post ACC TUS (experiment 2; n = 3); and

rs-fMRI from nine animals were acquired without stimulation (control; n = 9). All animals were purchased from a UK breeding center (Centre for Macaques, Porton Down, UK). All animals were socially housed and kept under a 12:12 light/dark cycle. No water or food regulation was needed for the conduction of this project. All procedures were conducted under licenses from the United Kingdom (UK) Home Office in accordance with The Animals (Scientific Procedures) Act 1986. In all cases they complied with the European Union guidelines (EU Directive 2010/63/EU).

All suitable animals available at the time of experimentation took part in this study. Accordingly, there was no pre-selection nor restriction for group allocation. No suitable and available datasets were excluded: data from all TUS sessions where the sonication could be focused on the target coordinates were included. Sample sizes could not be predetermined statistically in the absence of a prior literature reporting relevant expected effect sizes; instead we adopted sample sizes similar to those reported in previous publications detailing interventional macaque functional magnetic resonance imaging (fMRI) studies (O'Reilly et al., 2013). Data collection and analysis were not performed blind to the conditions of the experiments.

METHOD DETAILS

Ultrasound stimulation

A single-element ultrasound transducer (H115-MR, diameter 64 mm, Sonic Concept, Bothell, WA, USA) with a 51.74 mm focal depth was used with region-specific coupling cones filled with degassed water and sealed with a latex membrane (Durex) to assess TUS of amygdala (experiment 1) and ACC (experiment 2) (Figure 1). The ultrasound wave frequency was set to the 250 kHz resonance frequency and 30 ms bursts of ultrasound were generated every 100 ms (duty cycle 30%) with a digital function generator (Handyscope HS5, TiePie engineering, Sneek, the Netherlands). Overall, the stimulation lasted for 40 s. A 75-Watt amplifier (75A250A, Amplifier Research, Souderton, PA) was used to deliver the required power to the transducer. A TiePie probe (Handyscope HS5, TiePie engineering, Sneek, the Netherlands) connected to an oscilloscope was used to monitor the voltage delivered. The recorded peak-to-peak voltage was constantly maintained throughout the stimulation. Voltage values per session ranged from 128 to 134 V. It corresponded to a peak negative pressure ranging from 1.15 to 1.27 MPa respectively as measured in water with an in house heterodyne interferometer (Constans et al., 2017). The acoustic wave propagation of our focused ultrasound protocol was simulated at 130 V peak-to-peak voltage using finite element models of an entire monkey head to obtain estimates for the pressure amplitude, peak intensity, and spatial distribution (Constans et al., 2017). 3D maps of the skull were extracted from a monkey CT scan (0.36 mm isotropic resolution). Based on these numerical simulations, the maximum spatial peak pulse average intensity (I_{sppa}) in a focal region was estimated to be 64.9 W/cm^2 (spatial peak temporal average intensity (I_{spta}) = 19.5 W/cm^2) in the amygdala and 18.8 W/cm^2 (I_{spta} = 5.63 W/cm^2) in ACC with a maximum pressure of 1.44 MPa in amygdala and 0.78 MPa in ACC. One train was applied to each of the more laterally situated amygdalae but a single train was applied to the midline structure (ACC) in experiments 1 and 2 respectively.

Each individual animal's structural MR image was registered to its head with a frameless stereotaxic neuronavigation system (Rogue Research, Montreal, CA). By recording the positions of both the ultrasound transducer and the head with an infrared tracker it was then possible to co-register the ultrasound transducer with respect to the MRI scan of the brain to position the transducer over the targeted brain region, either ACC (MNI coordinates x = 0, y = 15, z = 6) or amygdala (MNI coordinates x = −10, y = 1, z = −11; x = 9, y = 1, z = −11). The ultrasound transducer / coupling cone montage was placed directly onto previously shaved skin on which conductive gel (SignaGel Electrode; Parker Laboratories Inc.) had been applied to ensure ultrasonic coupling between the transducer and the animal's head. In the non-stimulation condition (control), all procedures (anesthesia, pre-scan preparation, fMRI scan acquisition and timing), with the exception of actual TUS, mirrored the TUS sessions.

Macaque MRI acquisition

Resting state fMRI and anatomical MRI scans were collected under inhalational isoflurane anesthesia using a protocol which has previously proven successful (Neubert et al., 2015; Sallet et al., 2013) in preserving whole-brain functional connectivity as measured with BOLD signal. In the case of the TUS conditions, fMRI data collection began only after completion of the TUS train (delay between ultrasound stimulation offset and scanning onset: 37.5 minutes; SEM: 2.21 minutes). Anesthesia was induced using intramuscular injection of ketamine (10 mg/kg), xylazine (0.125-0.25 mg/kg), and midazolam (0.1 mg/kg). Macaques also received injections of atropine (0.05 mg/kg, intramuscularly), meloxicam (0.2 mg/kg, intravenously). The anesthetized animals were placed in a sphinx position and placed in a horizontal 3T MRI scanner with a full-size bore. Scanning commenced 1.53 hours (SEM: 4 minutes) and 2.38 hours (SEM: 4 minutes) after anesthesia induction in TUS and control sessions, respectively. In both cases data collection commenced when the clinical peak of ketamine had passed. Anesthesia was maintained, in accordance with veterinary recommendation, using the lowest possible concentration of isoflurane to ensure that macaques were anesthetized. The depth of anesthesia was assessed and monitored using physiological parameters (heart rate and blood pressure, as well as clinical checks before the scan for muscle relaxation). During the acquisition of the functional data, the inspired isoflurane concentration was in the range 0.8%–1.1%, and the expired isoflurane concentration was in the range 0.7%–1%. Isoflurane was selected for the scans as it has been demonstrated to preserve rs-fMRI networks (Neubert et al., 2015; Sallet et al., 2013; Vincent et al., 2007). Macaques were maintained with intermittent positive pressure ventilation to ensure a constant respiration rate during the functional scan, and respiration rate, inspired and expired CO_2, and inspired and expired isoflurane concentration were monitored and recorded using VitalMonitor software (Vetronic

Services Ltd.). Core temperature and peripheral capillary oxygen saturation (SpO_2) were also constantly monitored throughout the scan.

A four-channel phased-array coil was used for data acquisition (Dr. H. Kolster, Windmiller Kolster Scientific, Fresno, CA, USA). Whole-brain BOLD fMRI data were collected from each animal for up to 78 minutes. All fMRI data were collected using the following parameters: 36 axial slices; in-plane resolution, 2 × 2 mm; slice thickness, 2 mm; no slice gap; TR, 2000 ms; TE, 19 ms; 800 volumes per run. A minimum period of 10 days elapsed between sessions.

A structural scan (average over up to three T1-weighted structural MRI images) was acquired for each macaque in the same session as the functional scans, using a T1-weighted magnetization-prepared rapid- acquisition gradient echo sequence (0.5 × 0.5 × 0.5 mm voxel resolution).

Macaque MRI preprocessing

The preprocessing and analysis of the MRI data (Verhagen et al., 2019) was designed to follow the HCP Minimal Processing Pipeline (Glasser et al., 2013), using tools of FSL (Jenkinson et al., 2012; https://fsl.fmrib.ox.ac.uk/fsl/fslwiki), HCP Workbench (https://www.humanconnectome.org/software/connectome-workbench), and the Magnetic Resonance Comparative Anatomy Toolbox (MrCat; www.neuroecologylab.org).

The T1w images were processed using tools of FSL in an iterative fashion cycling through brain-extraction (BET), RF bias-field correction, and linear and non-linear registration (FLIRT and FNIRT) to the *Macaca mulatta* F99 atlas (Van Essen and Dierker, 2007). The application of robust and macaque-optimized versions of BET and FAST also provided segmentation into gray matter, white matter, and cerebral spinal fluid compartments. Segmentation of subcortical structures was obtained by registration to the D99 atlas (Reveley et al., 2017).

The first 5 volumes of the functional EPI datasets were discarded to ensure a steady RF excitation state. EPI timeseries were motion corrected using MCFLIRT. Given that the animals were anesthetized and their heads were held in a steady position, any apparent image motion, if present at all, is caused by changes to the B0 field, rather than by head motion. Accordingly, the parameter estimates from MCFLIRT can be considered to be 'B0-confound parameters' instead. Each timeseries was checked rigorously for spikes and other artifacts, both visually and using automated algorithms; where applicable slices with spikes were linearly interpolated based on temporally neighboring slices. This procedure identified an epoch with strong noise contributions at the end of the last run of a single rs-fMRI session following amygdala TUS. Accordingly, for one animal the last 254 volumes (out of a total of 2,400) were removed from further analysis. Brain extraction, bias-correction, and registration was achieved for the functional EPI datasets in an iterative manner, similar to the preprocessing of the structural images with the only difference that the mean of each functional dataset was registered to its corresponding T1w image using rigid-body boundary-based registration (FLIRT). EPI signal noise was reduced both in the frequency and temporal domain. First, the functional time series were high-pass filtered at 2000s. Temporally cyclical noise, for example originating from the respiratory apparatus, was removed using band-stop filters set dynamically to noise peaks in the frequency domain. Remaining temporal noise was described by the mean time course and the first two subsequent principal components of the white matter (WM) and cerebral spinal fluid (CSF) compartment (considering only voxels with a high posterior probability of belonging to the WM or CSF, obtained in the T1w image using FAST). The B0-confound parameter estimates were expanded as a second degree Volterra series to capture both linear and non-linear B0 effects. Together the WM and CSF expanded B0 confound parameters were regressed out of the BOLD signal for each voxel.

The cleaned time course was then low-pass filtered with a cut-off at 10 s. The cleaned and filtered signal was projected from the conventional volumetric representation (2mm voxels) to the F99 cortical surface (~1.4mm spaced vertices) using Workbench command "myelin-style" mapping, while maintaining the subcortical volumetric structures. The data was spatially smoothed using a 3mm FWHM Gaussian kernel, while taking into account the folding of the cortex and the anatomical boundaries of the subcortical structures. Lastly, the data were demeaned to prepare for functional connectivity analyses.

To represent subject effects, the timeseries from the three runs were concatenated to create a single timeseries per animal per intervention (control, ACC TUS, amygdala TUS). To represent group effects the run-concatenated timeseries of all animals were combined using a group-PCA approach (Smith et al., 2014) that was set to reduce the dimensionality of the data.

QUANTIFICATION AND STATISTICAL ANALYSIS

Macaque rs-fMRI connectivity analysis

Although the blood oxygen level dependent (BOLD) signal recorded with fMRI does not provide an absolute measure of activity it does provide a relative measure of activity change in relation to external events or activity recorded from other brain areas. This means that we cannot easily use BOLD to capture a measure such as activity in a brain area averaged over time. However, what we can do is to examine how BOLD responses in one area, such as the one that we are sonicating, relate to BOLD in another area using approaches similar to those employed previously (Margulies et al., 2009; Neubert et al., 2015; Sallet et al., 2013; Vincent et al., 2007).

To construct a region-of-interest (ROI) for ACC, a circle of 4mm radius was drawn on the cortical surface around the point closest to the average stimulation coordinate (Figure 1), in both the left and the right hemisphere. The same procedure was used to define other bilateral cortical regions of interest, based on literature coordinates (Neubert et al., 2015; Sallet et al., 2013; Neubert et al., 2014), to

serve as targets for the fingerprint and spatial extent analyses (Figure 2, sub-panel i). The amygdala ROI was constructed for each animal individually through non-linear registration of their T1w image to the D99 template and by subsequently resampling the (subcortical) D99 macaque atlas in native space (Reveley et al., 2017).

In order to make a statistical comparison of the functional coupling of the amygdala or ACC in the control and TUS conditions it is problematic to compare coupling at each and every other point in the brain because there is a risk of false positive effects if multiple comparisons are made. Given the limited sample sizes possible with non-human primate experiments, however, there is a risk of false negative results if stringent correction for multiple comparisons is undertaken at the whole-brain level. Indeed, here we avoid these pitfalls and reproduce whole-brain functional connectivity maps unthresholded to report on the full extent of the effects. Importantly, statistical inference was drawn on a limited set of regions beyond the amygdala known to be interconnected with macaque amygdala or ACC from anatomical tracing studies (Amaral and Price, 1984) and to exhibit, again in macaques, activity coupling with the amygdala under anesthesia (Neubert et al., 2015). An additional consideration was that some of the areas were connected to (Van Hoesen et al., 1993) and exhibited activity coupling with ACC (Neubert et al., 2015). Finally some areas, such as primary motor cortex (M1) and posterior intraparietal sulcus (pIPS) were chosen because, by contrast, they have limited connections and coupling with amygdala or ACC.

Coupling between the activity of each region of interest and the rest of the brain was estimated by calculating the Fisher's z-transformed correlation coefficient between each point in the ROI and all other datapoints. The resulting 'connectivity-maps' were averaged across all points in the ROI, across both hemispheres. Accordingly, the final maps represent the average coupling of a bilateral ROI with the rest of the brain. The fingerprints are obtained by extracting the average coupling with each target ROI and averaging across the two hemispheres.

To assess the impact of TUS not only for the target areas but across the whole brain, we indexed for every point in the brain its activity coupling with the same *a priori* defined constellation of regions used throughout the analyses (Figures 2G and 2H), but now excluding the sonicated areas. We quantify the impact of TUS by comparing for each point the average coupling with this set of regions in the control condition with the coupling observed following amygdala TUS and following ACC TUS. This approach resulted in two 'heat-maps' that show the peak location and extent of the brain activity impacted by TUS over amygdala and ACC.

Independently of the nature of the mechanisms underlying TUS (see Dallapiazza et al., 2018; Fomenko et al., 2018; Tyler et al., 2018), if TUS affects brain activity in a specific manner then it would be expected that the normal relationship seen at rest between the activity in the stimulated region and activity elsewhere will change. This does not mean that activity induced by TUS is diffused across the brain or that it is induced in one area and then "spreads" to others. It means simply that the relationship between activity in one area and another is changing. Measurements of activity throughout any area of tissue that is similarly affected by the TUS may become more highly correlated with one another. However, if the stimulated tissue becomes less responsive to other inputs from elsewhere in the brain then the relationship between activity in the stimulated region and elsewhere will decrease.

Macaque rs-fMRI statistical inference

Statistical inference on the fingerprints was performed using non-parametric permutation tests on cosine similarity metrics describing how similar or dissimilar pairs of fingerprints are (Mars et al., 2016; Verhagen et al., 2019). The cosine similarity metric considers the shape of the fingerprint as a whole (but not its mean amplitude) and performs one test per pair of fingerprints, negating the necessity for correcting for multiple comparisons across fingerprint targets. In contrast to conventional parametric tests, this approach does not rely on assumptions about the shape of the distribution but will acknowledge dependencies between target ROIs in the fingerprint; as such this approach will avoid inflation of type I error. For each test we ran 10,000 permutations across individual fMRI runs to accurately approximate with high accuracy the true probability of rejecting the null-hypothesis of permutable conditions in this sample.

To examine the spatial extent of the neuromodulatory impact of TUS on activity coupling we extracted for every point in the brain, both subcortically and on the cortical surface, its average coupling strength with the fingerprint targets (Figure 2, sub-panel i), excluding the amygdala and ACC. This approach allowed the creation of a quantified spatial map of the difference in average coupling between the control state and amygdala TUS, and between the control state and ACC TUS. For regions affected by TUS this difference will be large, while for all other regions this difference is close to zero.

Statistical inferences on the anesthesia levels and associated physiological parameters were drawn in the context of generalized linear mixed-effects (GLME) models. These models considered the intercept, the TUS condition (control, amygdala, ACC), and the resting-state fMRI run index (1, 2, or 3) as fixed effects and the intercept and slope grouped per animal as random effects with possible correlation between them (as implemented in MATLAB, Mathworks, Natick, USA). The models were assumed to adhere to a normal distribution of the data and were fitted using Maximum-Pseudo-Likelihood estimation methods where the covariance of the random effects was approximated using Cholesky parameterization. Statistical significance was set at $\alpha = 0.05$, two-tailed, and estimated using conventional analyses of variance (ANOVA).

DATA AND SOFTWARE AVAILABILITY

FSL can be downloaded from https://fsl.fmrib.ox.ac.uk/fsl/fslwiki. HCP Workbench can be downloaded from https://www.humanconnectome.org/software/connectome-workbench. For any information regarding MrCat please see http://www.rbmars.dds.nl/lab/toolbox.html; further inquiries can be directed to Lennart Verhagen (lennart.verhagen@psy.ox.ac.uk). All dedicated software tools are available at https://github.com/neuroecology/MrCat. The data reported in this paper is available from https://git.fmrib.ox.ac.uk/lverhagen/amygdala-acc-tus. For any further inquiries regarding the data, please contact Jérôme Sallet (jerome.sallet@psy.ox.ac.uk).

Neuron, Volume *101*

Supplemental Information

Manipulation of Subcortical and Deep Cortical Activity in the Primate Brain Using Transcranial Focused Ultrasound Stimulation

Davide Folloni, Lennart Verhagen, Rogier B. Mars, Elsa Fouragnan, Charlotte Constans, Jean-François Aubry, Matthew F.S. Rushworth, and Jérôme Sallet

SUPPLEMENTAL INFORMATION

Manipulation of subcortical and deep cortical activity in the primate brain using transcranial focused ultrasound stimulation

Davide Folloni, Lennart Verhagen, Rogier B. Mars, Elsa Fouragnan, Charlotte Constans, Jean-François Aubry, Matthew F.S. Rushworth, Jérôme Sallet

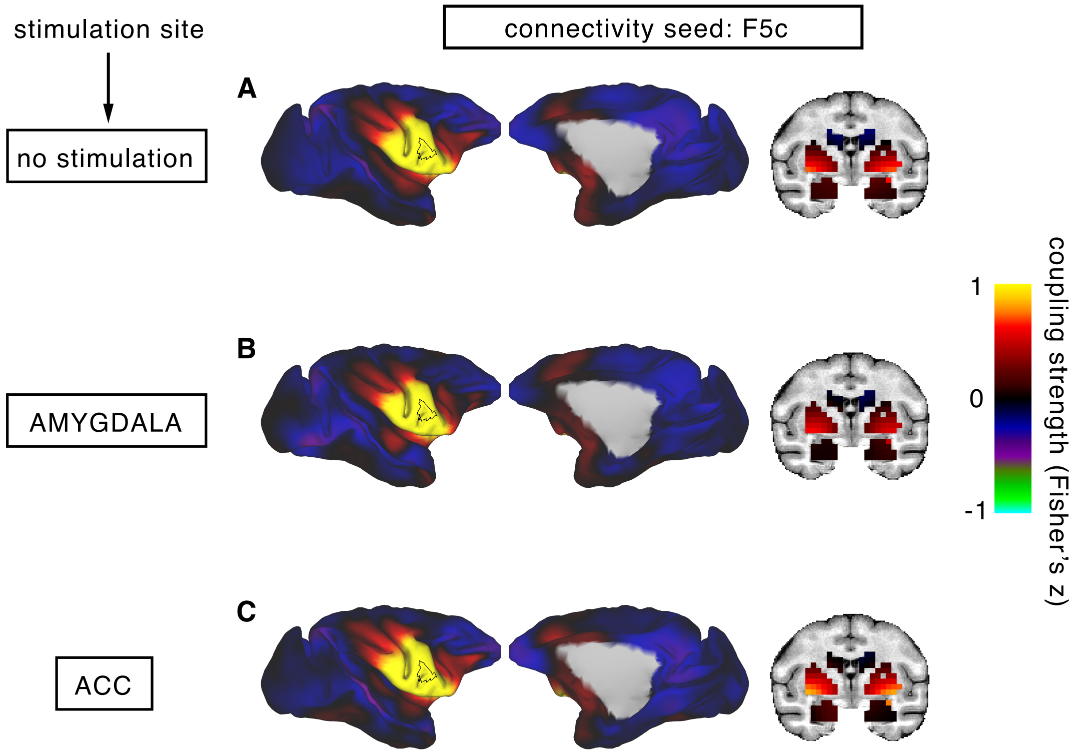

Figure S1. Whole-brain functional connectivity between stimulated and not stimulated areas with the rest of the brain, related to Figure 2

Panels A, B, and C show activity coupling between a control area, the caudal ventral premotor area F5c (outlined in black), and the rest of the brain in no stimulation/control condition (**A**), after amygdala TUS (**B**), and after ACC TUS (**C**). Hot colors indicate positive coupling (Fisher's z). Compared to a no stimulation condition (A), neither amygdala TUS nor ACC TUS (B, C) affected the whole-brain coupling activity of F5c which has weak anatomical connections with ACC and amygdala.

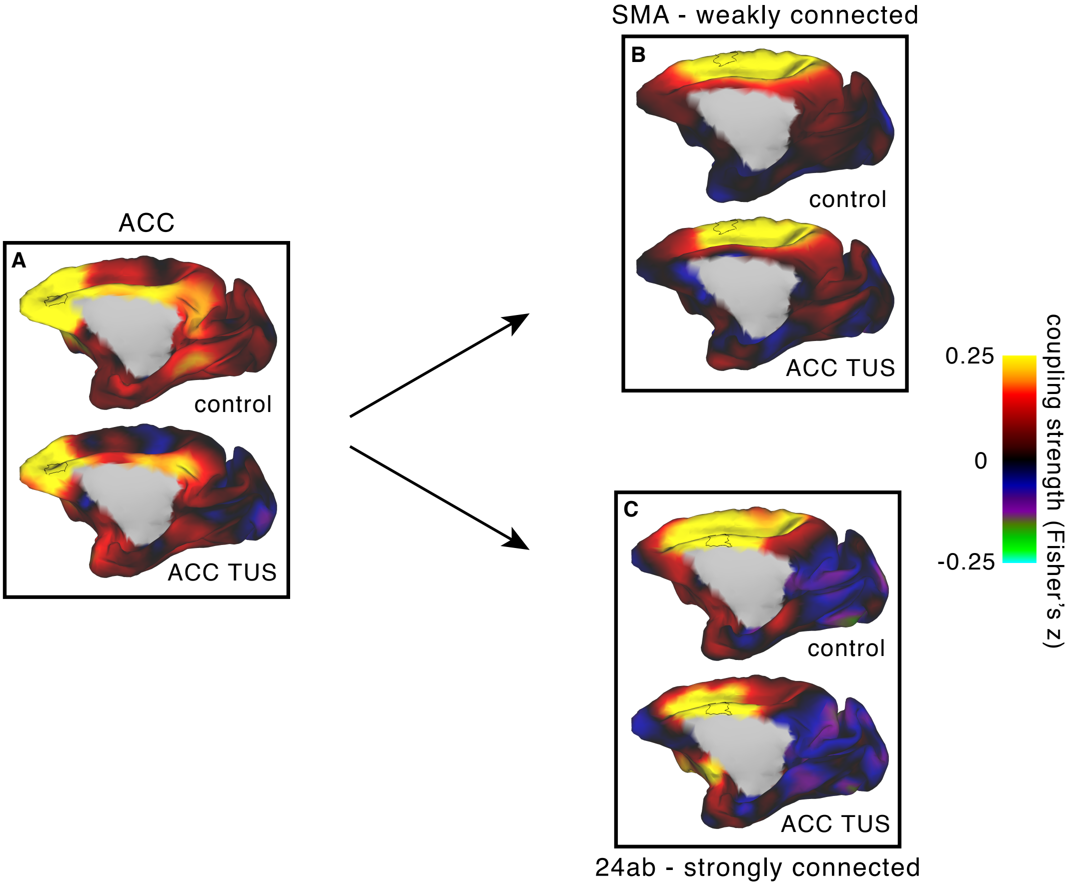

Figure S2. **Effects of TUS on regions outside the target area are mediated by the strength of anatomical connectivity rather than a result of spatial proximity, related to Figure 3**

Whole-brain activity coupling in control and ACC TUS conditions for ACC (**A**) and two areas at an equal Euclidian distance from the ACC target region but differing in their anatomical connectivity strength with the stimulated area: SMA (**B**) and area 24ab (**C**). Hot colors indicate positive coupling (Fisher's z). In SMA, which is less strongly connected to ACC, there were no changes (B) in the way in which its activity was coupled with that in other brain areas. This was in contrast with changes in activity coupling of area 24ab (C), which is more strongly connected to the ACC target compared to SMA (B).

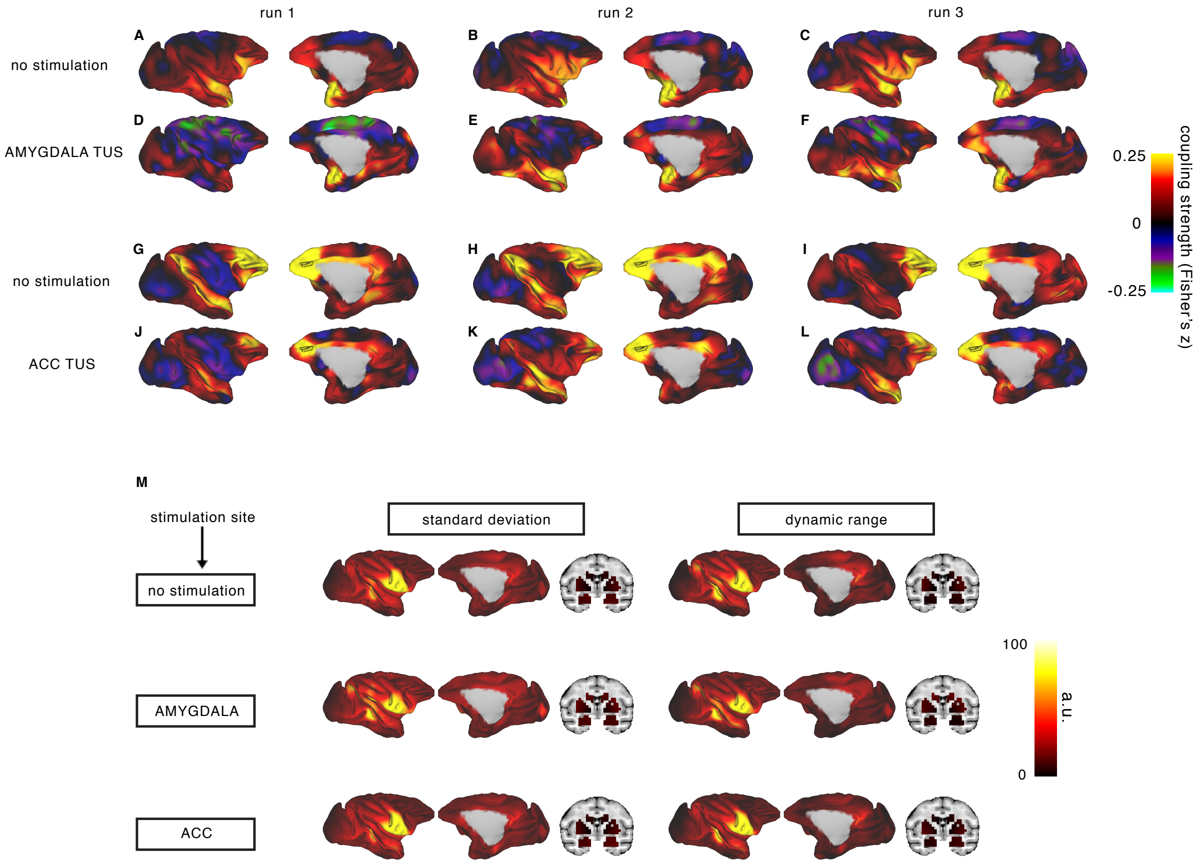

run 1
run 2
run 3
A
B
C
no stimulation
D
E
F
AMYGDALA TUS
G
H
I
no stimulation
J
K
L
ACC TUS
0.25
0
-0.25
coupling strength (Fisher's z)
M
stimulation site
standard deviation
dynamic range
no stimulation
AMYGDALA
ACC
100
0
a.u.

Figure S3. Temporal changes of TUS effects on amygdala and ACC functional coupling, related to Figures 2-3

Amygdala and ACC functional coupling across 3 consecutive runs is displayed after no stimulation (amygdala: **A**,**B**,**C**; ACC: **G**,**H**,**I**) and TUS (amygdala: **D**,**E**,**F**; ACC: **J**,**K**,**L**). TUS effects on the whole-brain coupling of each stimulated region persisted throughout the full length of scanning. Interestingly, TUS effects seem to show slightly decrease over the three runs with amygdala and ACC functional coupling resembling more their correspondent coupling in the no stimulation condition. (**M**) Gross indices of signal variability, time after induction, and physiological state related to anesthesia levels revealed no major effects of TUS condition. Temporal variability of the BOLD signal - a proxy for both the signal amplitude and the noise level - were similar across the control state and after either amygdala or ACC TUS. The anesthesia levels as indexed by expired isoflurane concentrations were well matched between conditions (see STAR Methods), the delay between sedation and data collection was on average comparable between amygdala TUS (2.00h) and the control (2.38h) sessions (amygdala TUS versus control: $F(1,34)=2.7654$, $p=0.10552$) but slightly shorter in the ACC TUS (1.44h; versus control: $F(1,31)=9.5537$, $p=0.0041946$). However, despite differences in the effects of ACC and amygdala TUS on functional connectivity, amygdala and ACC TUS sessions were very similar in duration ($F(1,19)=2.6863$, $p=0.11767$). Furthermore, there were no differences in any measurements of physiological parameters indexing the depth of anesthesia including expired isoflurane ($F(1.41)=0.37451$, $p=0.68995$), heart rate ($F(1,41)=1.8382$, $p=0.17198$), and respiration rate ($F(1,41)=0.032232$, $p=0.96831$) in control, amygdala, and ACC sessions.

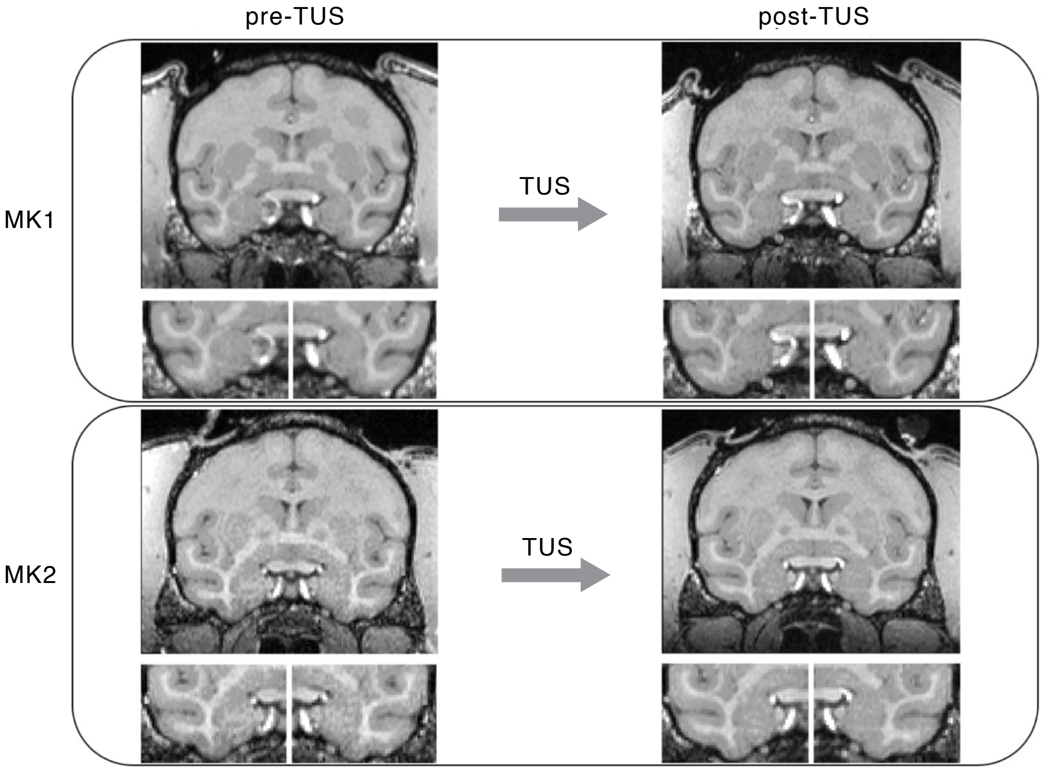

Figure S4. Lack of effects of TUS on brain tissue, related to STAR Methods

Coronal T1-weighted images collected before and after TUS (in this example targeted bilaterally to the amygdala) representing the stimulated brain area. Neither structural changes nor evidence of transient edema were found following TUS targeted to the amygdala bilaterally in two exemplar animals.
